# Protochlorophyllide synthesis by recombinant cyclases from eukaryotic oxygenic phototrophs and the dependence on Ycf54

**DOI:** 10.1042/BCJ20200221

**Published:** 2020-06-24

**Authors:** Guangyu E. Chen, C. Neil Hunter

**Affiliations:** Department of Molecular Biology and Biotechnology, University of Sheffield, Sheffield, U.K.

**Keywords:** chlorophyll, cyclase, photosynthesis, tetrapyrroles

## Abstract

The unique isocyclic E ring of chlorophylls contributes to their role as light-absorbing pigments in photosynthesis. The formation of the E ring is catalyzed by the Mg-protoporphyrin IX monomethyl ester cyclase, and the O_2_-dependent cyclase in prokaryotes consists of a diiron protein AcsF, augmented in cyanobacteria by an auxiliary subunit Ycf54. Here, we establish the composition of plant and algal cyclases, by demonstrating the *in vivo* heterologous activity of O_2_-dependent cyclases from the green alga *Chlamydomonas reinhardtii* and the model plant *Arabidopsis thaliana* in the anoxygenic photosynthetic bacterium *Rubrivivax gelatinosus* and in the non-photosynthetic bacterium *Escherichia coli*. In each case, an AcsF homolog is the core catalytic subunit, but there is an absolute requirement for an algal/plant counterpart of Ycf54, so the necessity for an auxiliary subunit is ubiquitous among oxygenic phototrophs. A C-terminal ∼40 aa extension, which is present specifically in green algal and plant Ycf54 proteins, may play an important role in the normal function of the protein as a cyclase subunit.

## Introduction

**Figure 1.**
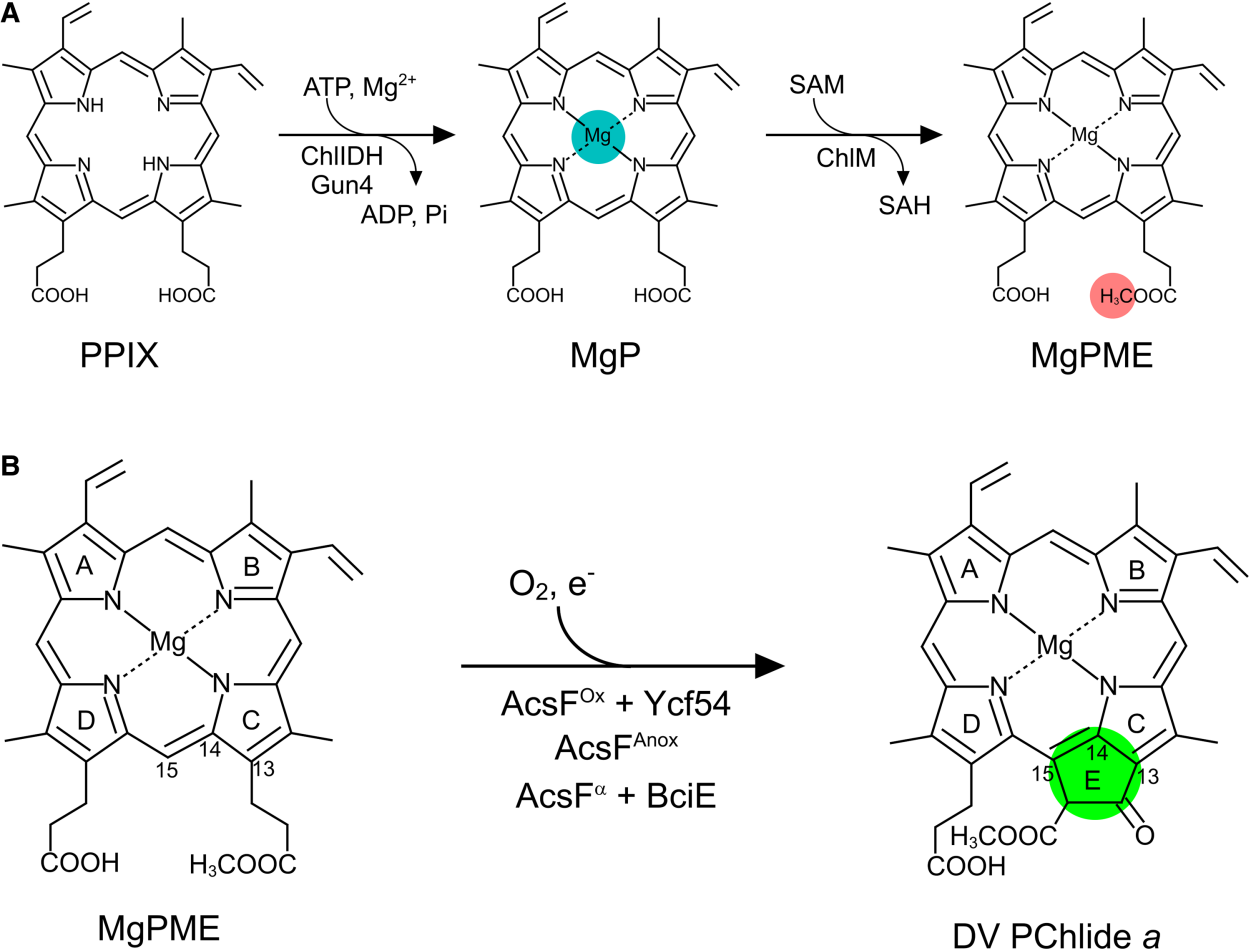
The early (B)Chl biosynthetic steps from PPIX to DV PChlide *a*. (**A**) Formation of MgPME from PPIX catalyzed by the magnesium chelatase (ChlIDH, Gun4) and the MgP methyltransferase (ChlM). ATP, adenosine triphosphate; ADP, adenosine diphosphate; Pi, inorganic phosphate; SAM, *S*-adenosine-l-methionine; SAH, *S*-adenosyl-l-homocysteine. (**B**) Formation of the isocyclic E ring (highlighted) of (B)Chl is catalyzed by three classes of O_2_-dependent cyclase: AcsF^Ox^ denotes AcsF from oxygenic phototrophs, which requires Ycf54; AcsF^Anox^ denotes AcsF from anoxygenic phototrophs excluding Alphaproteobacteria; and AcsF^α^ denotes alphaproteobacterial AcsF, which requires BciE. e^−^ represents the electron donor to the diiron center of AcsF. International Union of Pure and Applied Chemistry numbering of the relevant macrocycle carbons is indicated.

All chlorophototrophic organisms rely on the unique chemical properties of (bacterio)chlorophyll [(B)Chl] molecules for light harvesting and photochemical reactions, so the elucidation of the (B)Chl biosynthesis pathways is of great importance. In the common (B)Chl biosynthetic pathway, the least well-characterized step is the formation of the isocyclic E ring, via the oxidation and cyclization of the C13 methyl propionate group of Mg-protoporphyrin IX monomethyl ester (MgPME), producing 3,8-divinyl protochlorophyllide *a* (DV PChlide *a*). This step is catalyzed by two mechanistically unrelated cyclases: an O_2_-sensitive, radical SAM enzyme containing [4Fe–4S] and cobalamin cofactors [1] is encoded by the *bchE* gene in most anoxygenic phototrophic bacteria and some cyanobacteria [2], whereas an O_2_-dependent diiron monooxygenase is found in many purple bacteria, as well as cyanobacteria, algae and plants [3]. The catalytic subunit of the O_2_-dependent cyclase was first identified from the purple betaproteobacterium *Rubrivivax* (*Rvi.*) *gelatinosus* and named AcsF (aerobic cyclization system Fe-containing subunit) [4]. Following the identification of two auxiliary subunits, Ycf54 [5,6] and BciE, the O_2_-dependent cyclase has been delineated into three distinct classes: the Ycf54-dependent enzyme from oxygenic phototrophs, the AcsF-only enzyme in most anoxygenic phototrophs (e.g. in *Rvi. gelatinosus*) and the BciE-dependent alphaproteobacterial enzyme [3] (Figure 1B). The activity of the O_2_-dependent cyclase has been demonstrated in *Escherichia coli* using heterologously expressed enzymes from *Rvi. gelatinosus* and the cyanobacterium *Synechocystis* sp. PCC 6803 (hereafter *Synechocystis*) [7], thus completing the identification of the O_2_-dependent cyclase components in prokaryotic phototrophs.

Oxygenic phototrophs, in particular higher plants, rely solely on the O_2_-dependent cyclase for Chl biosynthesis. Early research work was focused on biochemical characterization of the enzyme using fractions from cucumber developing chloroplasts, resolving the enzyme into soluble and membrane components [8,9]; a similar subcellular distribution was found subsequently for the *Synechocystis* enzyme [10]. The model plant *Arabidopsis thaliana* (hereafter *Arabidopsis*) contains a single AcsF protein, CHL27 [11], a homolog of AcsF from *Rvi. gelatinosus* [4], whereas the green alga *Chlamydomonas reinhardtii* (hereafter *Chlamydomonas*) has two AcsF isoforms, CRD1 and CTH1. Genes encoding these isoforms are expressed reciprocally; CRD1 has a basal level of expression that increases under copper- or O_2_-deplete conditions, whereas CTH1 is produced under copper- and O_2_-replete conditions [12,13]. Similarly, *Synechocystis* also has two AcsF homologs, CycI and CycII; CycI is constitutively active under both aerobic and micro-oxic conditions, whilst CycII is additionally required under micro-oxic conditions [14,15]. AcsF proteins have been shown to be membrane-bound in various organisms [5,11,16,17]. In the search for the ‘missing’ soluble component, Ycf54/LCAA was identified as an auxiliary cyclase subunit from *Synechocystis* by *in vivo* pulldown experiments using FLAG-tagged CycI and CycII as bait [5], and from tobacco by screening antisense plants with Chl deficiency [6]. The key role of Ycf54 proteins in cyclase function is exemplified by its ubiquity in oxygenic phototrophs and the severe phenotypes exhibited by knockout mutants including impaired AcsF protein levels, accumulation of MgPME, and lowered amounts of DV PChlide *a* and Chl [5,6,18–21].

Despite this recent progress with the O_2_-dependent cyclase from purple bacteria and cyanobacteria, there has been no activity-based assignment of algal or plant cyclase components. We have shown previously that *Rvi. gelatinosus* and *E. coli* are suitable hosts for heterologous expression of cyclase components and demonstration of cyclase activity [3,7], and in this study we extend this approach to the O_2_-dependent cyclase subunits from *Synechocystis*, *Chlamydomonas* and *Arabidopsis*, representative of cyanobacteria, green algae and higher plants, respectively. The detection of *in vivo* cyclase activity from recombinant *Arabidopsis* and *Chlamydomonas* enzymes shows that the eukaryotic cyclase consists of AcsF and Ycf54 subunits and Ycf54 is, therefore, an essential component of cyclases in oxygenic phototrophs.

## Materials and methods

### Synthesized genes

Gene fragments encoding *Arabidopsis* CHL27 (AT3G56940.1), YCF54 (AT5G58250.1), *Chlamydomonas* CRD1(CHLREDRAFT_183476), CTH1 (CHLREDRAFT_205856), and CGL78 (CHLREDRAFT_162021) proteins, lacking the N-terminal chloroplast transit peptides predicted by the ChloroP 1.1 Server [22], were synthesized (Integrated DNA Technologies) with codons optimized for expression in *E. coli.* It is worth noting that, based on the sequence alignments of Ycf54 proteins and experimental tests, *Arabidopsis* YCF54 was assumed to contain a 72 aa chloroplast transit peptide instead of the predicted 80 aa. The nucleotide sequences of synthesized genes are listed in Supplementary Table S1.

### Plasmids and bacterial strains

The pBB[*gene*] plasmids were made by cloning the indicated *gene* fragment into the *Bgl*II/*Not*I or *Bgl*II/*Xho*I sites of pBBRBB-*Ppuf_843–1200_* [23]*.* The *Synechocystis cycI* and *cycI-ycf54* genes were amplified using the pK18[*cycI-ycf54*] plasmid [3] as a template. Overlap extension PCR was used to generate a *CHL27-YCF54* gene fragment with a ribosome binding site (TATAGGAGCTTGGATT) placed between the two genes. To apply the link and lock method [24], genes were first cloned individually into the *Nde*I/*Spe*I sites of a modified pET3a vector (containing an added *Spe*I site immediately upstream of the *BamH*I site). Then the genes were cut from the pET3a plasmids and adjoined consecutively in the described order using the procedures described previously [7]. Primers used in this study are listed in Supplementary Table S2. Plasmids were sequenced by Eurofins Genomics and are listed in Supplementary Table S3. *E. coli* strains were grown in LB medium and, if required, antibiotics were supplemented at 30 µg ml^−1^ for kanamycin and 100 µg ml^−1^ for ampicillin. *Rvi. gelatinosus* strains were grown in polypeptone-yeast extract-sodium succinate (PYS) medium [25] at 30°C and, when required, antibiotics were added at 40 µg ml^−1^ for rifampicin and 50 µg ml^−1^ for kanamycin. A spontaneous rifampicin-resistant mutant, Δ*bchE*Δ*acsF Rif^R^*, was isolated from the *Rvi. gelatinosus* Δ*bchE*Δ*acsF* [3] mutant and served as a platform strain to test foreign cyclases from *Synechocystis* and *Arabidopsis.* Cyclase genes were cloned into the expression vector pBBRBB-*Ppuf_843–1200_* to get the pBB[*gene*] plasmids, which were conjugated into the Δ*bchE*Δ*acsF Rif^R^* strain via the *E. coli* S17-1 strain. *E. coli* S17-1 cells containing the plasmid were grown in LB medium with 30 µg ml^−1^ kanamycin at 37°C for 24 h and 30 µl of the resulting culture were mixed with *Rvi. gelatinosus* cells harvested from 30 ml culture and resuspended in 100 µl of LB medium. The mating mixture was placed onto a well-dried LB agar medium and incubated at 30°C overnight before streaking out onto PYS agar medium with 50 µg ml^−1^ kanamycin and 40 µg ml^−1^ rifampicin to select for transconjugants. Bacterial strains used in this study are listed in Supplementary Table S3.

### Phenotypic analysis of *Rvi. gelatinosus* strains

*Rvi. gelatinosus* strains were grown in 10 ml PYS medium, supplemented with 50 µg ml^−1^ kanamycin if the strain contains a pBBRBB-*puf*_843–1200_-based plasmid, in 50 ml Falcon tubes at 30°C with shaking at 175 rpm for 2 days. Harvested cells were either used for recording whole-cell absorption spectra or subjected to pigment extraction. Absorption spectra were recorded on a Cary 60 UV–Vis spectrophotometer with cells resuspended in 60% (w/v) sucrose, and normalized by the absorbance at 950 nm, followed by subtraction of the Δ*bchE*Δ*acsF Rif^R^* spectrum to correct for light scattering. Pigment extraction and subsequent HPLC analysis were performed as described previously [26].

### *In vivo* cyclase assay in *E. coli*

*E. coli* C43(DE3) [27] was transformed with the pET3a-based plasmids and selected on LB agar supplemented with 100 µg ml^−1^ ampicillin. A single colony was inoculated to 10 ml LB medium with 100 µg ml^−1^ ampicillin and grown overnight at 37°C with shaking at 220 rpm. The next day, 50 µl of the resulting culture were used to inoculate 10 ml of LB medium with 100 µg ml^−1^ ampicillin and grown at 37°C for 3 h. Then isopropyl-β-D-thiogalactopyranoside (IPTG) was added at 0.5 mM to induce gene expression. δ-Aminolevulinic acid (ALA) and Mg^2+^ (equimolar mixture of MgCl_2_ and MgSO_4_) were also added at 10 mM. The culture was incubated at 30°C with shaking at 175 rpm for 24 h before pigment extraction. Pigment extraction and high-performance liquid chromatography (HPLC) analysis were conducted as described previously [7] with slight modifications. The column was a Phenomenex Luna C18(2) reversed-phase column (particle size, 5 µm; pore size, 100 Å; 250 × 4.6 mm). The wash with 100% solvent B was shortened from 15 to 5 min. Elution was additionally monitored by fluorescence at 640 nm excited at 440 nm.

### SDS–PAGE and immunodetection

*E. coli* strains containing the pET3a-based plasmids were incubated and induced as for the *in vivo* cyclase assay. After 5 h induction cells were harvested and resuspended in 50 mM Tris–HCl, pH 8.0, 5 mM EDTA, supplemented with Proteinase Inhibitor Cocktail (Sigma–Aldrich), and lysed by sonication on ice (3 ×  20 s with 20 s resting between cycles). The cell lysate was clarified by centrifugation at 3000×***g*** at 4°C for 5 min to remove unbroken cells and cell debris, followed by SDS–PAGE analysis using NuPAGE 12% Bis-Tris Protein Gels (Thermo Fisher Scientific). Protein bands were transferred to a polyvinylidene difluoride (PVDF) membrane, which was probed with specific primary antibodies raised against *Arabidopsis* CHL27 (Agrisera), YCF54 (Agrisera) and *Synechocystis* Ycf54 [5], and then with a secondary anti-rabbit antibody conjugated with horseradish peroxidase (Sigma–Aldrich). Chemiluminescent signal was developed using the WESTAR SUN enhanced chemiluminescence substrate (Cyanagen) and detected by an Amersham Imager 600 (GE Healthcare).

## Results

### Complementation of a *Rvi. gelatinosus* Δ*bchE*Δ*acsF* mutant with *Synechocystis* and *Arabidopsis* cyclase genes

**Figure 2.**
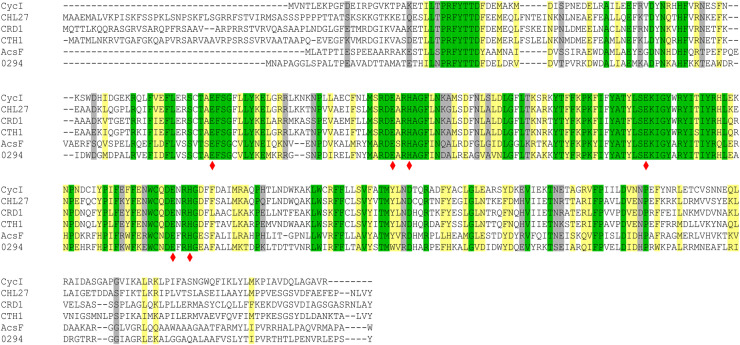
Amino acid sequence alignments of AcsF proteins. Sequences are those from *Synechocystis* sp. PCC 6803 (CycI, BAA16583), *Arabidopsis thaliana* (CHL27, NP_191253), *Chlamydomonas reinhardtii* (CRD1, XP_001692557; CTH1, XP_001691047), *Rubrivivax gelatinosus* IL144 (AcsF, BAL96694) and *Rhodobacter sphaeroides* 2.4.1 (0294, abbreviated for RSP_0294, YP_353369). Conserved, highly similar and similar residues are highlighted in green, yellow and gray, respectively. The putative diiron binding ligands are marked with red diamonds.

**Figure 3.**
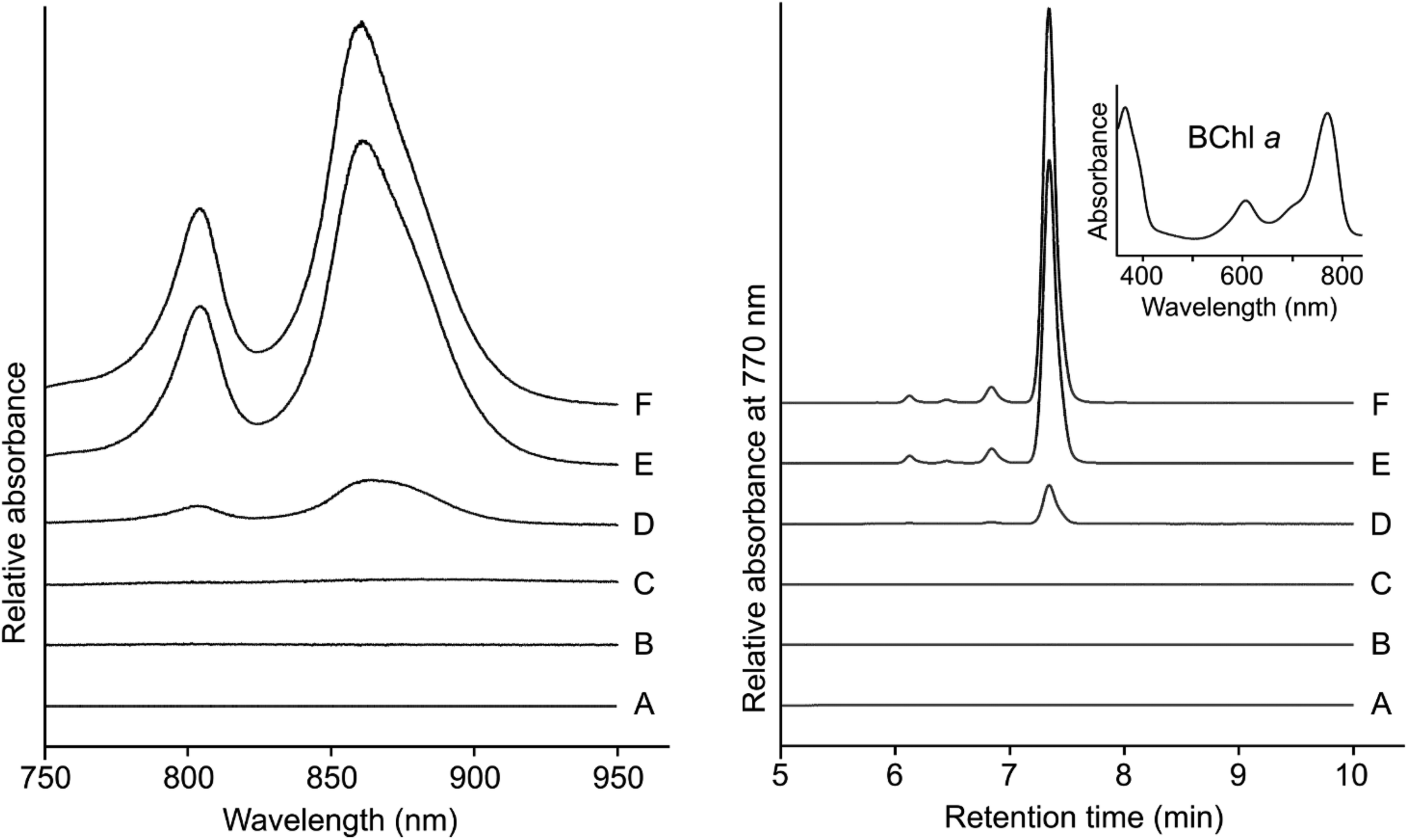
Heterologous activity of *Synechocystis* and *Arabidopsis* cyclases in *Rvi. gelatinosus* Cyclase genes were tested in the *Rvi. gelatinosus* Δ*bchE*Δ*acsF Rif^R^* mutant by expression from a plasmid vector: (**A**) no plasmid, (**B**) pBB[*cycI*], (**C**) pBB[*CHL27*], (**D**) pBB[*CHL27-YCF54*], (**E**) pBB[*acsF*] and (**F**) pBB[*cycI-ycf54*]. *Left*, whole-cell absorbance spectra of the described strains, normalized and corrected for light scattering. *Right*, HPLC elution profiles of pigment extracts from the described strains standardized by cell number. The major peak in the traces was identified to be BChl *a* by the retention time and absorbance spectrum (*inset*).

The catalytic subunit of O_2_-dependent cyclase, AcsF, containing a signature diiron binding motif (E-x_n_-E-x-x-H-x_n_-E-x_n_-E-x-x-H), is classified as a membrane-bound carboxylate diiron protein [28] (Figure 2). We used a Δ*bchE*Δ*acsF Rif^R^* mutant of the facultative phototroph *Rvi. gelatinosus*, which lacks both the O_2_-sensitive and -dependent cyclase enzymes [3], as a background strain for assaying plasmid-borne *Synechocystis cycI* and *ycf54* genes, or genes encoding the *Arabidopsis* CHL27 and YCF54 proteins. Whole-cell absorption spectra of transconjugant strains showed that neither CycI nor CHL27 was functional but when partnered with Ycf54 or YCF54, respectively, each was able to replace the missing native cyclase and restore the assembly of photosynthetic complexes to variable levels (Figure 3). It is striking that the activity of the *Synechocystis* CycI-Ycf54 pair matched that of the native AcsF protein (Figure 3). We further analyzed the accumulation of BChl *a* in these strains by HPLC and the detected level is in good agreement with the abundance of photosynthetic complexes revealed by the corresponding whole-cell absorption spectra (Figure 3).

### *In vivo* activity of the recombinant *Synechocystis* and *Arabidopsis* cyclases in *E. coli*

**Figure 4.**
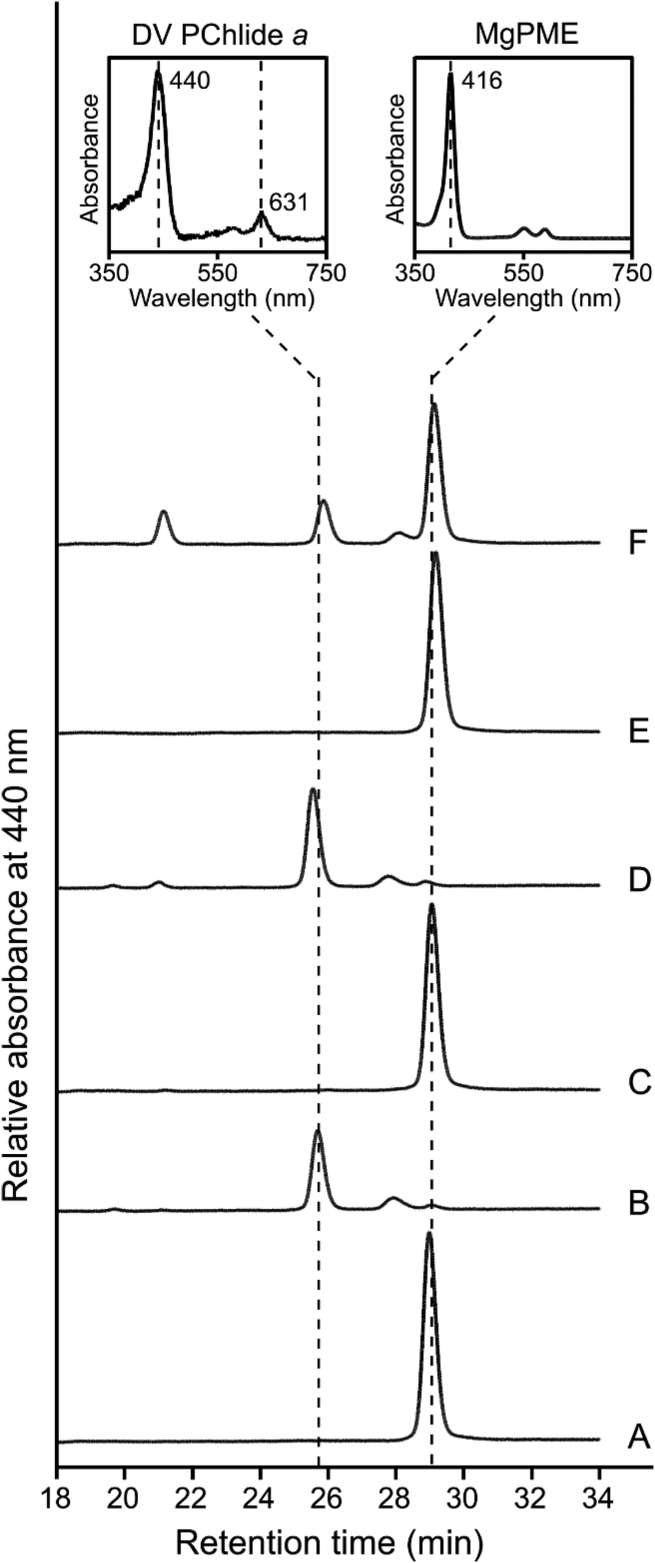
HPLC analysis of pigments extracted from *E. coli* strains expressing *Synechocystis* and *Arabidopsis* cyclases. *E. coli* strains contained (**A**) IM (*chlI-chlD-chlH-gun4-chlM*), (**B**) IA (*chlI-chlD-chlH-gun4-chlM-acsF*), (**C**) IM-*cycI*, (**D**) IM-*cycI-ycf54*, (**E**) IM-*CHL27* and (**F**) IM-*CHL27-YCF54* plasmids. Protein expression was induced with IPTG, and ALA and Mg^2+^ were supplemented to boost the production of cyclase substrate, MgPME. Pigments were extracted from the same number of cells except for the strains containing IA and IM-*cycI-ycf54* plasmids, for which only 1/10th of cells were used. Elution of cyclase substrate and product was monitored by absorbance at 440 nm. Retention times and spectra (*inset*) of peaks are used to identify pigment species.

The *Synechocystis* CycI-Ycf54 and *Arabidopsis* CHL27-YCF54 pairs were clearly active in the purple phototrophic bacterium *Rvi. gelatinosus*. Next, we adopted a previously reported strategy [7] to demonstrate their activity in the non-photosynthetic bacterium *E. coli*. Cyclase encoding genes were cloned into the pET3a-based plasmid IM [7], which contains genes encoding the first two enzymes of the Chl pathway that convert native, endogenous protoporphyrin IX (PPIX) to Mg-protoporphyrin IX (MgP), then to the cyclase substrate, MgPME (Figure 1A). These constructs were transformed into the *E. coli* C43(DE3) strain, followed by *in vivo* cyclase assays. Pigments from the harvested *E. coli* cells were extracted and analyzed by HPLC with elution profiles monitored by absorbance, and also fluorescence for a higher sensitivity for detecting Chl biosynthesis intermediates. In the absence of a functional cyclase, only MgPME (Soret band at 416 nm; emission maximum at 593 nm) was detected, which eluted at ∼29 min (Figure 4). The *E. coli* strains expressing *Rvi. gelatinosus* AcsF (plasmid IA), *Synechocystis* CycI-Ycf54 (IM-*cycI-ycf5*4) and *Arabidopsis* CHL27-YCF54 (IM-*CHL27-YCF54*), accumulated DV PChlide *a* (Soret/Q_y_ bands at 440 and 631 nm, respectively; emission maximum at 639 nm) as represented by a peak at ∼26 min, which is accompanied by a decreased level of MgPME (Figure 4). *Arabidopsis* CHL27-YCF54 was less active than the *Rvi. gelatinosus* and *Synechocystis* enzymes, leaving a considerable amount of MgPME substrate not converted to product (Figure 4).

### The *Chlamydomonas* AcsF isoform CRD1, but not CTH1, is functional when expressed in *E. coli*

**Figure 5.**
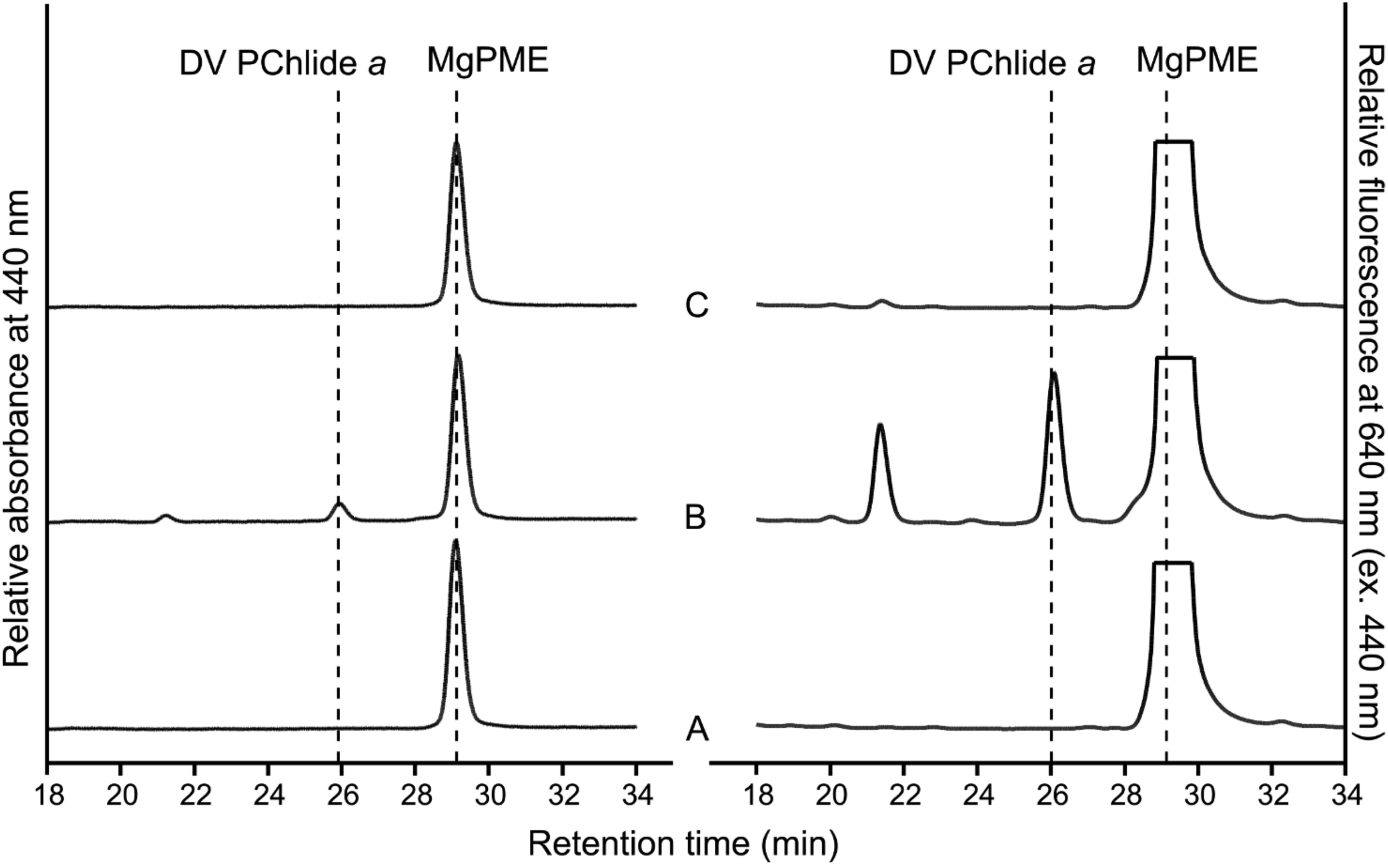
HPLC analysis of pigments extracted from *E. coli* strains expressing *Chlamydomonas* cyclases. Analysis of *E. coli* strains containing (**A**) IM-*CRD1*, (**B**) IM-*CRD1-CGL78* and (**C**) IM-*CTH1-CGL78* plasmids. Pigments were extracted from the same number of cells. Elution of cyclase substrate and the product was monitored by absorbance at 440 nm and fluorescence at 640 nm excited at 440 nm.

Unlike the single AcsF found in *Arabidopsis*, the green alga *Chlamydomonas* contains two AcsF isoforms, CRD1 and CTH1 [13]. Genes encoding CRD1, CTH1 and the *Chlamydomonas* Ycf54 protein, CGL78, were synthesized and cloned into the IM plasmid [7] for *in vivo* cyclase assay in *E. coli*. HPLC analysis of pigments extracted from the assays reveals that CRD1 was functional only when CGL78 was co-expressed (Figure 5). In the current test system, no activity was detected for CTH1 even in the presence of CGL78 (Figure 5).

### *Arabidopsis* and *Chlamydomonas* Ycf54 proteins differ from their *Synechocystis* homolog

**Figure 6.**
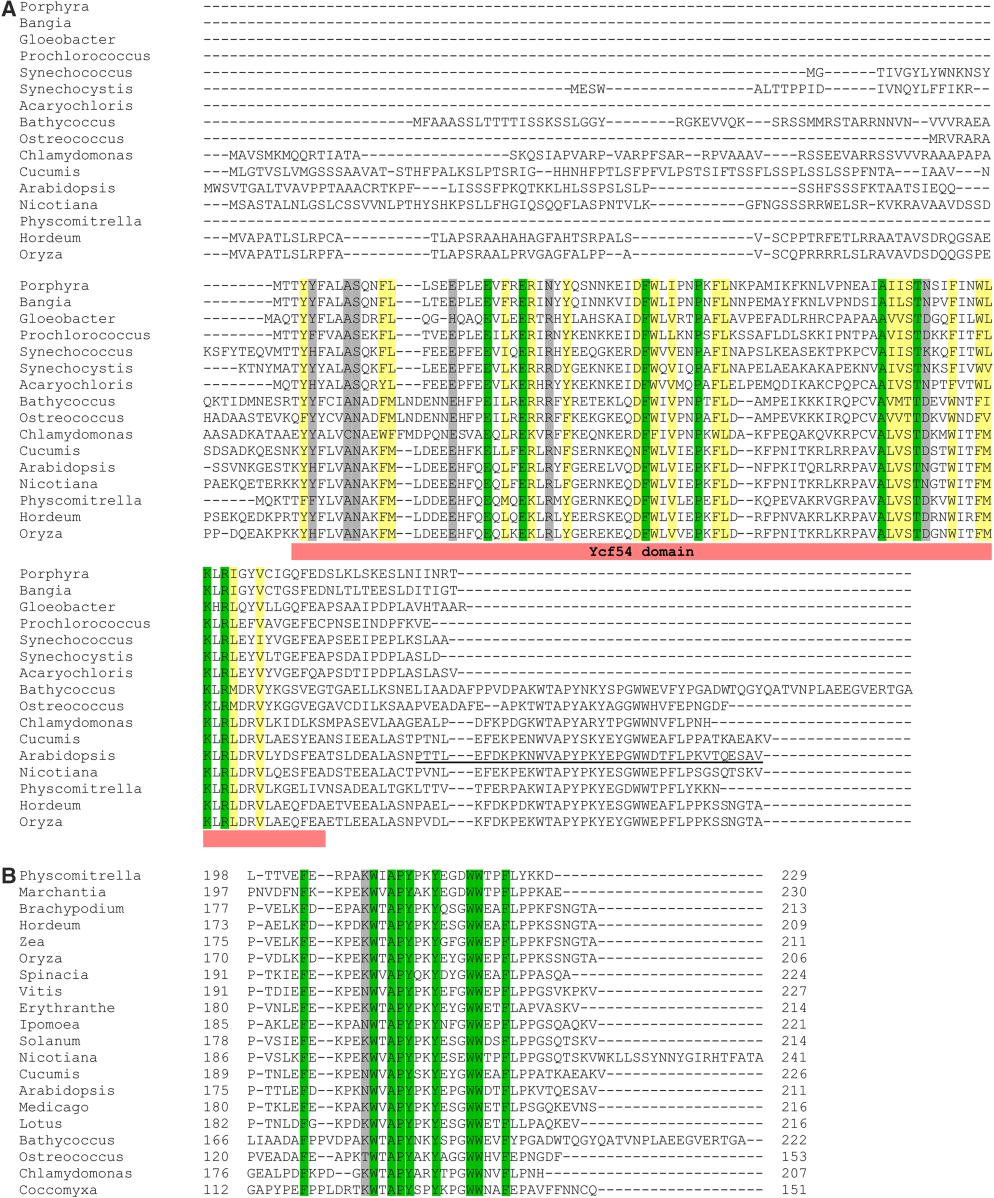
Amino acid sequence alignments of Ycf54 proteins. Conserved, highly similar and similar residues are highlighted in green, yellow and gray, respectively. (**A**) Sequences are those from the primordial cyanobacterium *Gloeobacter violaceus* PCC 7421 (NP_923828); cyanobacteria, *Prochlorococcus marinus* MED4 (CAE19565), *Synechococcus* sp. PCC 7002 (ACA98109), *Synechocystis* sp. PCC 6803 (BAA16792), *Acaryochloris marina* MBIC11017 (ABW27358); red algae, *Porphyra purpurea (*NP_053814), *Bangia fuscopurpurea* (AKE98807); green algae, *Bathycoccus prasinos* (XP_007514179), *Ostreococcus tauri* (XP_022839105), *Chlamydomonas reinhardtii* (XP_001691121); the moss *Physcomitrella patens* (XP_001756877); higher plants, *Cucumis sativus* (XP_004139926), *Arabidopsis thaliana* (NP_200633), *Nicotiana tobacum* (XP_016480530), *Hordeum vulgare* L. cv. Bonus (BAJ91312), *Oryza sativa* L. ssp. *japonica* (XP_015628146). The conserved Ycf54 domain is indicated. The C-terminal 37 aa sequence deleted in the YCF54* mutant is underlined. (**B**) Sequence alignments showing the conservation of the C-terminal extensions present specifically in green algal and plant Ycf54 proteins. Additional green algal and plant Ycf54 sequences are included, which are from the green alga *Coccomyxa subellipsoidea* C-169 (XP_005642819); the liverwort *Marchantia polymorpha* (PTQ33664); higher plants, *Brachypodium distachyon* (XP_003557990), *Zea mays* (NP_001131876), *Spinacia oleracea* (XP_021852323), *Vitis vinifera* (XP_010650790), *Erythranthe guttata* (XP_012838088), *Ipomoea nil* (XP_019188624), *Solanum lycopersicum* (XP_004240451), *Medicago truncatula* (XP_013460499), *Lotus japonicus* (AFK37846). Full-length sequences were used for alignments but for clarity, only the C-terminal extension regions with the residue range indicated, are shown.

**Figure 7.**
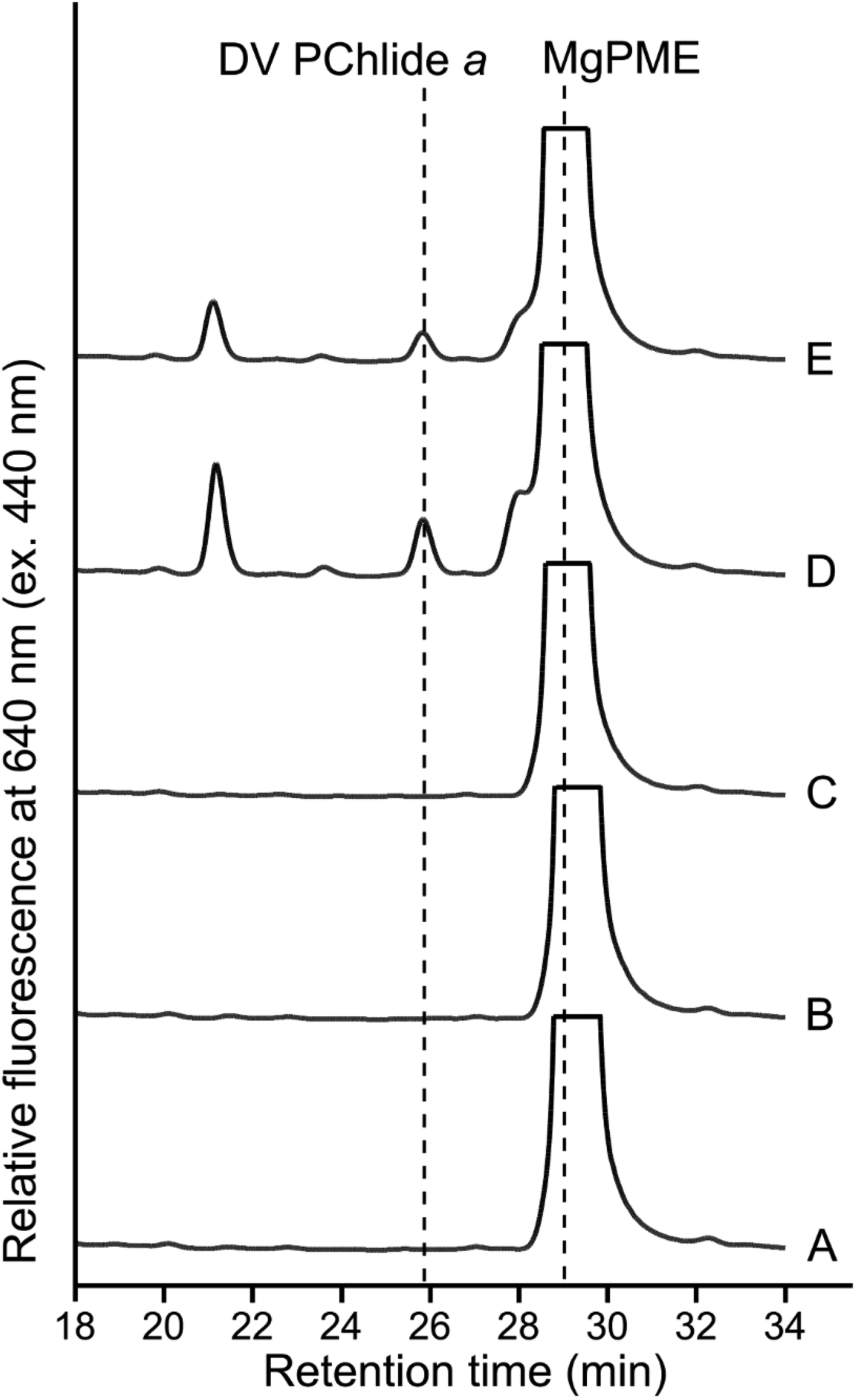
HPLC analysis of pigments extracted from *E. coli* strains expressing YCF54* and swapped *ycf54* genes. *E. coli* strains contained (**A**) IM-YCF54*, (**B**) IM-*CHL27-ycf54*, (**C**) IM-*CRD1-ycf54*, (**D**) IM-*cycI-YCF54* and (**E**) IM-*cycI-CGL78* plasmids. Pigments were extracted from the same number of cells. Elution of cyclase substrate and the product was monitored by fluorescence at 640 nm excited at 440 nm.

Our *in vivo* cyclase assays using enzymes from representative species of cyanobacteria, green algae and plants, reveal the universal requirement of Ycf54 for cyclase activity among oxygenic phototrophs. Sequence alignments of Ycf54 proteins clearly identify a conserved Ycf54 domain (designated PF10674/DUF2488 in the Pfam database; Figure 6A). The alignments also reveal C-terminal extensions present only in green algal and plant Ycf54 proteins (Figure 6A), containing nine conserved residues (F180, W186, A188, P189, Y190, Y193, W197, W198 and W201; numbering in *Arabidopsis* YCF54, NP_200633) among these species (Figure 6B). To investigate the potential role of the C-terminal extension, we truncated the *Arabidopsis YCF54* gene to generate a YCF54* mutant, with the C-terminal 37 aa sequence removed. We also swapped Ycf54 proteins between *Arabidopsis*, *Chlamydomonas* and *Synechocystis* cyclases to check whether Ycf54 is functional with a foreign AcsF protein. CHL27-YCF54* behaved essentially the same as CHL27 alone in the *in vivo* cyclase assays, indicating the inactive nature of YCF54* (Figure 7). The *Synechocystis* Ycf54 protein was unable to replace the *Arabidopsis* or *Chlamydomonas* counterpart whereas *Arabidopsis* YCF54 and *Chlamydomonas* CGL78 supported some cyclase activity (detectable by fluorescence) when partnered with *Synechocystis* CycI (Figure 7). These results suggest green algal and plant Ycf54 proteins differ from the proteins found in other organisms, probably by their C-terminal extensions.

### Expression levels of recombinant cyclase subunits in *E. coli*

**Figure 8.**
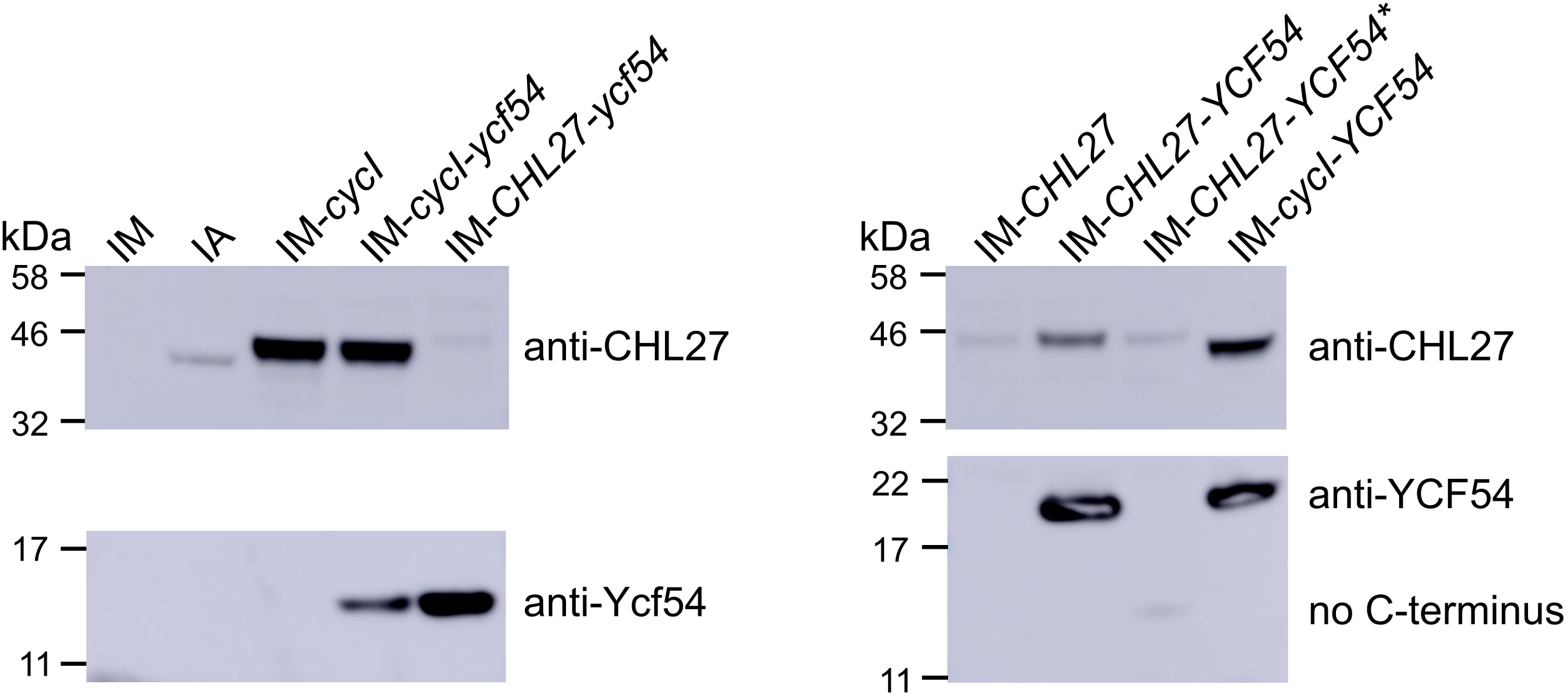
Immunodetection of recombinant cyclase subunits expressed in *E. coli*. *E. coli* strains containing the indicated plasmids were grown, induced as for the *in vivo* cyclase assay and cells were harvested after 5 h induction. Clarified cell lysates were analyzed by SDS–PAGE with loading standardized by OD_600_, followed by transfer to a PVDF membrane for immunodetection. Cyclase subunits were detected by using antibodies raised against *Arabidopsis* CHL27, YCF54 and *Synechocystis* Ycf54.

Our *in vivo* cyclase assays demonstrate the cyclase from oxygenic phototrophs is dependent on the Ycf54 protein for activity. To check whether Ycf54 affects the expression/stability of AcsF when expressed in *E. coli*, we detected the level of AcsF and Ycf54 proteins in clarified cell lysates using specific antibodies. The commercially available anti-CHL27 antibody also reacts with *Synechocystis* CycI but does not react with *Chlamydomonas* CRD1 or CTH1 so the immunodetection was limited to *Arabidopsis* and *Synechocystis* cyclases. In contrast with the indistinguishable CycI levels with and without Ycf54, the CHL27 level was clearly dependent on its native interacting partner YCF54 (Figure 8). The absence or C-terminal truncation of YCF54 significantly decreased the abundance of CHL27, which could not be rectified by the presence of *Synechocystis* Ycf54 (Figure 8). Additionally, the C-terminal truncation seems to destabilize YCF54, and/or affect the interaction between YCF54 and its antibody as YCF54* produced a much weaker signal than YCF54 (Figure 8).

## Discussion

The enigma of the subunit composition of the O_2_-dependent cyclase has been clarified by the discovery of three distinct classes of the enzyme among photosynthetic organisms [3]. Genetic complementation experiments conducted in the *Rvi. gelatinosus* cyclase mutant revealed the equivalence between the three classes of the enzyme, as indicated in Figure 1B. The next advance involved demonstration of heterologous cyclase activity in *E. coli*, with two of the three classes of cyclase, from *Rvi. gelatinosus* and *Synechocystis* [7], essentially completing the long endeavour to find the ‘missing’ cyclase components. It is widely accepted that cyanobacteria are evolutionarily related to the chloroplasts of algae and plants, endorsed by the conservation of photosynthetic components and processes between cyanobacteria and eukaryotic phototrophs. This conservation also exists in the O_2_-dependent cyclase as indicated by the shared subunits, AcsF and Ycf54, so the likelihood that eukaryotic cyclase would require extra subunits was low. In this study, we show that, indeed, no extra cyclase-specific subunits are required, and heterologously expressed *Arabidopsis* CHL27 and YCF54 function as an active cyclase in *Rvi. gelatinosus* (Figure 3) and *E. coli* (Figure 4). We did not detect any cyclase activity of *Chlamydomonas* CTH1 co-expressed with CGL78 in *E. coli* (Figure 5), possibly because CTH1 was not expressed or it was expressed but unstable in *E. coli.* Alternatively, the activity of CTH1 may depend on specific copper and/or oxygen levels that differ from those in the *E. coli* cellular context since it has been documented that CTH1 is produced under copper- and oxygen-replete conditions in *Chlamydomonas* [12,13]. Nevertheless, we were able to demonstrate the *in vivo* cyclase activity of *Chlamydomonas* CRD1 and CGL78 produced in *E. coli* (Figure 5). A reductase is likely required, but *E. coli* can provide this function for a variety of bacterial, algal and plant cyclases, and *Rvi. gelatinosus* can also supply reductase activity for a cyclase from a cyanobacterium [3] and a plant (Figure 3). Thus, this study has defined the cyclase-specific subunits of the eukaryotic cyclase.

At this point, it is necessary to mention the cyclase studies conducted with barley *xantha-l* and *viridis-k* mutants, which suggested that the enzyme in this organism contains at least one soluble and two membrane-bound components, of which one is encoded by the *Xantha-l* gene, an *acsF* orthologue, and the other could be defective in the *viridis-k* mutants [16]. The suggestion that barley Ycf54 is a membrane-associated cyclase component increased the potential number of components to at least four [29]. Analysis of barely mutants is a powerful tool, but it is likely to detect lesions in serially linked processes because Chl biosynthesis is co-ordinated with photosystem biogenesis through multiple regulation checkpoints such as the initiating magnesium chelatase and the light-dependent PChlide oxidoreductase steps, and potentially the O_2_-dependent cyclase step [30–32]. Thus, cyclase-deficient phenotypes are not necessarily due to lack of a cyclase subunit but can be a product of perturbed regulation or unstable cyclase components or even shortage of co-substrates/cofactors. Moreover, various factors can affect cyclase activity as indicated by the studies showing that the NADPH-dependent thioredoxin reductase and 2-Cys peroxiredoxins [33,34], and the thylakoid plastoquinone pool, are linked with cyclase activity [33,35].

Analogous to other diiron enzymes, the diiron center of cyclase needs to be reduced from +3 to +2 during the catalytic cycle by a reductant or an electron donor, which is believed to be NADPH as suggested by *in vitro* cyclase assays conducted with chloroplast/plastid fractions from plants and *Chlamydomonas* [8,10,16] and with *Synechocystis* cell extracts [10]. However, both AcsF and Ycf54 lack an apparent NAD(P)H-binding motif, implying that the direct electron donor is yet to be determined. As a cellular electron currency, NADPH is utilized to reduce many redox-active components, and the biochemical fractions used for *in vitro* cyclase assays probably contained some of these redox-active components, one of which could be the direct electron donor to the diiron center of cyclase. Herbst *et al*. [20] recently proposed that the plastidal ferredoxin-NADPH reductase (FNR) could be involved in reducing the cyclase based on the interaction between FNR1 and YCF54, and the disturbed cyclase activity that resulted from FNR1 deficiency. In oxygenic phototrophs FNR utilizes the electrons from photosynthetically reduced ferredoxin (Fd) to produce NADPH, and when there is a shortage of reduced Fd, such as in the dark, the enzyme can also catalyze the reverse reaction. Reduced Fd serves as the electron donor to a few redox enzymes including some involved in (B)Chl biosynthesis: Fd-dependent 8-vinyl reductase [36], dark-operative PChlide oxidoreductase [37], chlorophyllide oxidoreductase [38] and Chl *a* oxygenase [39]. In particular, some diiron enzymes use reduced Fd as the reductant as in the case for plant stearoyl-acyl carrier protein Δ^9^ desaturase [40] and *p-*aminobenzoate *N-*oxygenase [41]. To identify the direct electron donor to cyclase, future work should test reduced Fd as well as other types of reductants using purified cyclase subunits rather than undefined complex cellular fractions. Considering the heterologous activity of cyclase in non-pigmented *E. coli* [7] (Figures 4 and 5), it is predictable that the as-yet unidentified reductant and its potentially existing oxidoreductase are generic and shared with other metabolic processes rather than being specific to the cyclase reaction.

Our work further confirms that Ycf54 is an authentic cyclase subunit in oxygenic phototrophs. In their native hosts, Ycf54 is required for the accumulation of AcsF as demonstrated in *Synechocystis* [5,18,19], tobacco [6] and *Arabidopsis* [20] and its interaction with AcsF, mediated by the conserved R82 residue that forms part of a positively charged patch on Ycf54, is required for optimal PChlide formation as shown in *Synechocystis* [19]. The dependency of AcsF accumulation on Ycf54 was also found for the *Arabidopsis* enzyme when heterologously expressed in *E. coli*, but not for the *Synechocystis* cyclase as indicated by the unaffected level of CycI in the absence of Ycf54 (Figure 8). These results cannot rule out the possibility that Ycf54 plays a role in the assembly of the diiron center of CycI, which requires future characterization of purified CycI from a Ycf54-minus background. It is also possible that Ycf54 plays a role in substrate delivery/channelling or, alternatively, electron transfer during the catalytic cycle; *in vitro* biochemical assays using purified AcsF and Ycf54 proteins will be required to investigate a catalytic role for Ycf54. In addition, swapping Ycf54 proteins between different cyclase enzymes revealed the *Synechocystis* protein differs from its *Arabidopsis* and *Chlamydomonas* homologs as it was unable to stabilize CHL27 (Figure 8) and its co-expression with CHL27 or CRD1 did not result in a functional cyclase (Figure 7). This difference may be explained by the C-terminal extension, which is present only in green algal and plant Ycf54 proteins (Figure 6A), and which is highly conserved in eukaryotic phototrophs (Figure 6B). Despite its small size, ∼40 aa, this C-terminal extension is significant in the context of the ∼15 kDa Ycf54 protein and its removal abolishes cyclase activity (Figure 7), the basis for which appears to be the absence of any YCF54 protein, and also a significantly lowered level of CHL27 (Figure 8). We propose that the C-terminal extension plays a role in the normal function of green algal and plant YCF54 and CHL27 proteins. It has been reported that *Arabidopsis* and barley YCF54 proteins form oligomers [6,29] whereas the 1.3 Å structure of the *Synechocystis* protein indicates that it is monomeric [19]. The C-terminal extension may stimulate and/or maintain the oligomerisation of Ycf54 through hydrophobic interactions between its highly conserved residues, of which 7 out of 9 are aromatic (Figure 6B). Alternatively, the C-terminal extension may be involved in the interaction between green algal and plant Ycf54 and AcsF proteins. Our work also suggests that future studies involving the heterologous activity of CHL27, and possibly CRD1, in *E. coli* will benefit from co-expression of their cognate Ycf54 proteins.

## Abbreviations

MgP: Mg-protoporphyrin IX
PPIX: protoporphyrin IX
PVDF: polyvinylidene difluoride
PYS: polypeptone-yeast extract-sodium succinate
