# Supplementary material for: Protochlorophyllide synthesis by recombinant cyclases from eukaryotic oxygenic phototrophs and the dependence on Ycf54
Source: Biochem J. 2020 Jun 24;477(12):2313–25. doi: 10.1042/BCJ20200221 (PMC7319587; doi:10.1042/BCJ20200221)
Supplement: Supplementary Tables S1-S3 [file BCJ-477-2313-s1.pdf]

SUPPLEMENTARY MATERIALS

Table S1. Nucleotide sequences of synthesized genes used in this study

Gene	Sequence (5’-3’)
CHL27	ATGAGCGCCAGCTCTTCTCCACCTCCTCCAACTACTGCCACGAGCAAGTCAAAGAAAGGTAC<br>AAAAAAGGAGATACAAGAATCCCTGTTGACGCCTCGTTTTTACACTACCGATTTTGAGGAGA<br>TGGAGCAGTTGTTTAACACTGAGATAAACAAAAATCTTAATGAAGCCGAGTTTGAAGCTCTT<br>TTACAAGAGTTTAAAACCGATTATAACCAAACTCATTTCGTCCGGAATAAGGAGTTCAAGGA<br>AGCAGCTGACAAACTTCAGGGTCCTTTACGTCAAATTTTCGTGGAATTCTTGGAGAGATCAT<br>GTACCGCTGAGTTTTCGGGATTCTTGCTGTACAAGGAACTTGGTCGGCGCCTGAAGAAAACC<br>AACCCTGTAGTTGCAGAGATTTTCTCCTTGATGAGCCGCGACGAAGCAAGACACGCGGGCTT<br>TTTAAATAAAGGTTTGAGCGATTTTAATTTGGCGCTTGATTTAGGGTTTCTTACCAAAGCCC<br>GCAAATATACTTTTTTCAAACCGAAATTCATTTTTTACGCGACTTATCTTAGCGAAAAGATA<br>GGTTACTGGCGCTACATAACAATTTATCGCCACTTAAAAGAAAATCCGGAGTTCCAGTGTTA<br>TCCGATCTTCAAATACTTCGAGAACTGGTGCCAAGATGAAAATCGGCATGGCGACTTCTTCT<br>CCGCCCTGATGAAGGCGCAGCCTCAATTTTTAAATGACTGGCAGGCAAAACTGTGGAGCCGC<br>TTCTTCTGCTTGAGCGTATACGTCACGATGTACTTAAATGATTGCCAGCGTACAAATTTTTA<br>TGAGGGTATTGGGCTTAACACCAAAGAGTTTGATATGCATGTTATAATCGAGACGAACCGGA<br>CTACGGCTAGAATATTTCCTGCCGTATTAGACGTGGAGAATCCAGAATTCAAACGCAAACTT<br>GATCGTATGGTGGTCTCGTATGAGAAGTTACTTGCGATAGGAGAGACAGATGATGCAAGTTT<br>CATCAAAACATTGAAACGTATCCCGTTGGTCACATCATTGGCCTCCGAGATCCTGGCGGCAT<br>ACTTGATGCCGCCAGTAGAATCCGGTTCTGTAGATTTTGCTGAGTTCGAGCCAAATTTGGTC<br>TACTAA
YCF54	ATGTCAACGAAATATCATTTTTTGGTAGCAAATGCCAAATTTATGTTGGATGAAGAGGAGCA<br>TTTCCAAGAGCAATTATTTGAGCGCCTTCGGTATTTCGGAGAGCGGGAACTGGTACAAGATT<br>TTTGGCTGGTCATTGAACCCAAATTCCTGGATAATTTCCCCAAGATAACCCAACGCTTGCGG<br>CGGCCAGCCGTAGCGCTTGTCAGCACTAATGGAACGTGGATCACCTTTATGAAGCTGCGTCT<br>GGACCGGGTGCTGTACGACTCGTTCGAAGCCACATCGTTAGATGAGGCGCTTGCGAGTAATC<br>CTACCACGCTTGAATTTGATAAGCCTAAGAATTGGGTAGCGCCGTATCCGAAGTACGAGCCA<br>GGCTGGTGGGATACCTTTTTGCCGAAGGTCACGCAAGAGTCCGCCGTCTAA
CRD1	ATGGTACAAGCAAGCGCGGCCCCGCTTAACGACGGTTTGGGGTTCGAAACGATGCGTGACGG<br>TATCAAAGTGGCCGCTAAGGAAACGTTGCTTACACCACGGTTTTATACGACGGACTTTGACG<br>AAATGGAGCAACTGTTCTCTAAGGAAATTAACCCAAACCTTGACATGGAAGAGCTGAACGCA<br>TGTCTTAATGAATTCCGTAACGATTACAATAAGGTTCACTTCGTCCGGAATGAAACGTTTAA<br>AGCCGCTGCGGACAAGGTAACAGGGGAGACCAGACGTATATTCATTGAGTTCCTTGAGCGGT<br>CCTGCACGGCAGAGTTTTCGGGCTTCTTGTTATATAAAGAGTTGGCTCGGCGGATGAAAGCC<br>AGCAGCCCGGAGGTAGCTGAGATGTTCTTGCTTATGTCCCGGGACGAGGCCCGTCATGCAGG<br>GTTTCTTAACAAGGCTTTGTCGGATTTTAACCTTGCCTTAGACCTTGGGTTTCTTACAAAGA<br>ATAGAACCTACACGTACTTTAAACCGAAATTTATCATTTATGCTACATTTTTAAGCGAAAAG<br>ATAGGGTACTGGAGATATATAACCATATATCGGCACCTGCAACGTAATCCGGACAATCAGTT<br>CTATCCCCTTTTTGAATACTTCGAAAACTGGTGCCAGGATGAAAACCGCCACGGTGATTTCC<br>TGGCAGCATGCCTTAAAGCGAAGCCCGAACTTTTGAACACCTTCGAAGCAAAGCTGTGGAGC<br>AAGTTTTTTTGCTTAAGCGTATATATTACAATGTATTTGAACGATCACCAACGCACCAAATT<br>CTATGAATCTCTTGGGTTGAATACTCGTCAATTCAACCAACATGTCATTATAGAAACCAACC<br>GCGCTACCGAAAGATTGTTTCCAGTCGTGCCTGACGTTGAAGATCCGAGATTTTTCGAAATC<br>CTTAATAAAATGGTGGATGTGAATGCCAAGCTGGTAGAGTTAAGTGCCTCGTCCAGCCCACT<br>TGCTGGACTTCAAAAGTTACCCCTTTTAGAGCGGATGGCCTCGTACTGCTTACAACTTTTGT<br>TTTTTAAGGAAAAGGACGTCGGATCGGTAGATATCGCCGGGAGTGGCGCTTCGAGAAACTTA<br>GCTTACTAA
CTH1	ATGGTGGCGGCAACAGCCGCGCCACAGGAAGTAGAAGGTTTTAAAGTTATGCGCGACGGCAT<br>TAAAGTGGCCTCCGACGAAACGCTTTTAACACCCCGTTTCTATACCACTGATTTTGACGAGA<br>TGGAGCGTCTTTTTAGTCTTGAGTTGAACAAGAATATGGACATGGAAGAGTTCGAAGCCATG<br>TTGAATGAATTTAAGTTGGATTATAATCAACGCCACTTTGTTCGCAATGAGACGTTTAAAGA<br>AGCGGCGGAAAAGATCCAAGGACCCACACGTAAAATCTTTATTGAGTTTCTTGAGCGTTCCT<br>GTACAGCAGAGTTCTCTGGATTTTTACTGTATAAGGAACTGGGACGTCGCCTGAAAGCTACG<br>AACCCAGTAGTAGCCGAAATTTTCACGCTTATGTCGCGCGACGAAGCACGTCATGCTGGTTT<br>TTTGAATAAAGCGATGTCCGATTTTAATTTGGCACTTGACTTAGGTTTCTTGACTAAGAACC<br>GTAAATATACTTTCTTCAAACCTAAATTCATCTTTTACGCCACATACTTATCCGAGAAAATC

	GGTTATTGGCGCTATATCAGTATCTACCGCCACCTGCAGCGCAATCCAGACAACCAATTGTA<br>CCCCTTATTCGAATATTTTGAGAACTGGTGTCAAGACGAAAACCGTCATGGTGACTTTTTCA<br>CTGCCGTCCTGAAAGCACGCCCGGAAATGGTCAATGATTGGGCAGCGAAACTGTGGTCACGC<br>TTCTTCTGCCTTAGTGTATACATTACGATGTACTTGAATGATCACCAACGTGACGCATTTTA<br>TAGCTCCCTGGGGCTGAACACAACGCAATTTAATCAACACGTGATCATTGAAACTAATAAAT<br>CGACAGAGCGTATTTTCCCTGCTGTGCCAGATGTGGAAAATCCTGAGTTTTTTCGCCGTATG<br>GACTTGTTGGTGAAATACAACGCACAACTGGTGAACATTGGCTCAATGAACTTGCCTTCTCC<br>CATTAAAGCAATTATGAAAGCACCTATCTTAGAACGCATGGTCGCGGAAGTCTTTCAAGTAT<br>TTATTATGACGCCGAAAGAGAGCGGTTCGTATGATCTTGATGCCAACAAGACAGCTCTTGTA<br>TATTAA
CGL78	ATGCCTGCTCCAGCAGCTGCTTCAGCCGATAAAGCAACAGCGGCCGAATACTACGCCCTTGT<br>CTGCAACGCCGAATGGTTCTTCATGGACCCGCAAAACGAGTCGGTTGCTGAACAATTGAGAG<br>AGAAGGTAAGATTCTTCAAAGAGCAGAACAAAGAACGGGATTTCTTCATCGTGCCAAATCCC<br>AAGTGGCTGGACGCCAAGTTCCCGGAACAGGCTAAGCAAGTCAAAAGACCATGCGTAGCGCT<br>GGTCAGTACCGACAAAATGTGGATTACATTCATGAAGTTGCGCTTGGACCGTGTGTTGAAGA<br>TCGACTTAAAAAGTATGCCAGCGAGCGAGGTCCTTGCGGCTGGGGAAGCATTGCCGGATTTC<br>AAGCCGGACGGAAAGTGGACAGCGCCGTATGCCAGATACACTCCCGGTTGGTGGAATGTATT<br>TCTGCCTAATCATTAA

Table S2. Primers used in this study

Primer	Sequence (5'-3')
cycIBglII_F	GAGTCTAGATCTATGGTTAATACCCTCGAAAAGCCC
cycINotI_R	GAGTCTGCGGCCGCTTAGCGCACAGCTCCAGCCAAC
ycf54NotI_R	GAGTCTGCGGCCGCCTAATCCAGGGATGCAAGGGGGT
CHL27BglII_F	GAAAGATCTATGAGCGCCAGCTCTTCTCC
CHL27XhoI_R	GAACTCGAGTTAGTAGACCAAATTTGGCTCGAA
CHL27rbsYCF54_F	AGCCAAATTTGGTCTACTAATATAGGAGCTTGGATTATGTCAACGAAATATCATT
CHL27rbsYCF54_R	AATGATATTTCGTTGACATAATCCAAGCTCCTATATTAGTAGACCAAATTTGGCT
YCF54XhoI_R	GAACTCGAGTTAGACGGCGGACTCTTGCG
CHL27NdeI_F	GGAACATATGAGCGCCAGCTCTTCTCC
CHL27SpeI_R	GGAAACTAGTTTAGTAGACCAAATTTGGCTCG
YCF54NdeI_F	GGAACATATGTCAACGAAATATCATTTTTTGGTA
YCF54SpeI_R	GGAAACTAGTTTAGACGGCGGACTCTTGCG
YCF54truncSpeI_R	GGAAACTAGTTTAATTACTCGCAAGCGCCTC
CRD1NdeI_F	GGAACATATGGTACAAGCAAGCGCGGCCC
CRD1SpeI_R	GGAAACTAGTTTAGTAAGCTAAGTTTCTCGAAG
CTH1NdeI_F	GGAACATATGGTGGCGGCAACAGC
CTH1SpeI_R	GGAAACTAGTTTAATATACAAGAGCTGTCTTGT
CGL78NdeI_F	GGAACATATGCCTGCTCCAGCAGCTGCTT
CGL78SpeI_R	GGAAACTAGTTTAATGATTAGGCAGAAATACAT

Table S3. Strains and plasmids described in this study

**Strain/Plasmid**	**Description**	**Source**
E. coli		
JM109	Cloning strain for plasmid constructs	Promega
S17-1	Conjugation strain for pBBRBB-$Ppuf_{843–1200}$ constructs	[1]
C43(DE3)	Expression strain for *in vivo* cyclase assays	[2]
Rvi. gelatinosus		
IL144	WT strain	S. Nagashima*
Δ*bchE*Δ*acsF*	Unmarked deletion mutant of *bchE* and *acsF* in WT	[3]
Δ*bchE*Δ*acsF* Rif^R	Spontaneous rifampicin resistant mutant isolated from Δ*bchE*Δ*acsF*	This study
**Plasmid**		
pBBRBB-$Ppuf_{843–1200}$	Expression vector carrying the 843–1200 region of the *puf* promoter of *Rba. sphaeroides*, Km^R	[4]
pBB[*acsF*]	*Rvi. gelatinosus acsF* gene cloned into BglII/NotI sites of pBBRBB-$Ppuf_{843–1200}$	[5]
pBB[*cycI*]	*Synechocystis cycI* gene cloned into BglII/NotI sites of pBBRBB-$Ppuf_{843–1200}$	This study
pBB[*cycI-ycf54*]	*Synechocystis cycI-ycf54* genes cloned into BglII/NotI sites of pBBRBB-$Ppuf_{843–1200}$	This study
pBB[*CHL27*]	Synthesized gene encoding *Arabidopsis* CHL27 cloned into BglII/XhoI sites of pBBRBB-$Ppuf_{843–1200}$	This study
pBB[*CHL27-YCF54*]	Synthesized genes encoding *Arabidopsis* CHL27 and YCF54 cloned into BglII/XhoI sites of pBBRBB-$Ppuf_{843–1200}$	This study
pET3a	Expression vector carrying T7 promoter, Amp^R	Novagen
IM	*Synechocystis chlI, chlD, chlH, gun4* and *chlM* genes cloned into pET3a sequentially using link and lock method	[5]
IA	*Rvi. gelatinosus acsF* gene cloned into IM	[5]
IM-*cycI*	*Synechocystis cycI* gene cloned into IM	[5]
IM-*cycI-ycf54*	*Synechocystis ycf54* gene cloned into IM-*cycI*	[5]
IM-*CHL27*	Synthesized gene encoding *Arabidopsis* CHL27 cloned into IM	This study
IM-*CHL27-YCF54*	Synthesized gene encoding *Arabidopsis* YCF54 cloned into IM-*CHL27*	This study

IM-*CRD1*	Synthesized gene encoding *Chlamydomonas* CRD1 cloned into IM	This study
IM-*CRD1-CGL78*	Synthesized gene encoding *Chlamydomonas* CGL78 cloned into IM-*CRD1*	This study
IM-*CTH1*	Synthesized gene encoding *Chlamydomonas* CTH1 cloned into IM	This study
IM-*CTH1-CGL78*	Synthesized gene encoding *Chlamydomonas* CGL78 cloned into IM-*CTH1*	This study
IM-*CHL27-ycf54*	*Synechocystis ycf54* gene cloned into IM-*CHL27*	This study
IM-*CRD1-ycf54*	*Synechocystis ycf54* gene cloned into IM-*CRD1*	This study
IM-*cycI-YCF54*	Synthesized gene encoding *Arabidopsis* YCF54 cloned into IM-*cycI*	This study
IM-*cycI-CGL78*	Synthesized gene encoding *Chlamydomonas* CGL78 cloned into IM-*cycI*	This study
IM-*CHL27-YCF54**	Synthesized gene encoding *Arabidopsis* YCF54 with C-terminal 37 aa truncated (YCF54*) cloned into IM-*CHL27*	This study

*Research Institute for Photosynthetic Hydrogen Production, Kanagawa University, Yokohama, Japan.

†School of Life Sciences, Arizona State University, AZ 85281.

References

1 Simon, R., Priefer, U. and Puhler, A. (1983) A broad host range mobilization system for *in vivo* genetic engineering: transposon mutagenesis in gram negative bacteria. *Nat. Biotechnol.* **1**, 784–791

2 Miroux, B. and Walker, J. E. (1996) Over-production of proteins in *Escherichia coli*: mutant hosts that allow synthesis of some membrane proteins and globular proteins at high levels. *J. Mol. Biol.* **260**, 289–298

3 Chen, G. E., Canniffe, D. P. and Hunter, C. N. (2017) Three classes of oxygen-dependent cyclase involved in chlorophyll and bacteriochlorophyll biosynthesis. *Proc. Natl. Acad. Sci. U.S.A.* **114**, 6280–6285

4 Tikh, I. B., Held, M. and Schmidt-Dannert, C. (2014) BioBrick compatible vector system

for protein expression in *Rhodobacter sphaeroides*. *Appl. Microbiol. Biotechnol.* **98**, 3111–3119

Chen, G. E., Canniffe, D. P., Barnett, S. F. H., Hollingshead, S., Brindley, A. A., Vasilev, C., Bryant, D. A. and Neil Hunter, C. (2018) Complete enzyme set for chlorophyll biosynthesis in *Escherichia coli*. *Sci. Adv.* **4**, eaaq1407
